# (2*E*,4*E*)-Ethyl 5-(2,4-di­chloro­phenyl­sulfon­yl)penta-2,4-dienoate

**DOI:** 10.1107/S1600536813021429

**Published:** 2013-08-07

**Authors:** U. Sankar, V. Sabari, S. Mahalakshmi, K. K. Balasubramanian, S. Aravindhan

**Affiliations:** a Department of Chemistry, Pachaiyappa’s College, Chennai 600 030, India; b Department of Physics, Presidency College, Chennai 600 005, India; c Department of Chemistry, B.S. Abdur Rahman University, Chennai 600 048, India

## Abstract

In the title compound, C_13_H_12_Cl_2_O_4_S, both C=C double bonds adopt an *E* conformation. The S atom has a distorted tetra­hedral geometry with bond angles ranging from 103.03 (12) to 118.12 (13)°. The eth­oxy­carbonyl group is disordered over two sets of sites, with site-occupancy factors of 0.739 (11) and 0.261 (11). In the crystal, C—H⋯O inter­actions link the mol­ecules into chains mol­ecules running parallel to the *a* axis.

## Related literature

For the biological activity of phenyl­sulfonyl-containing compounds, see: De Benedetti *et al.* (1985 ▶). For related structures, see: Li (2011 ▶); Sankar *et al.* (2012 ▶); Chakkaravarthi *et al.* (2008 ▶); Rodriguez *et al.* (1995 ▶).
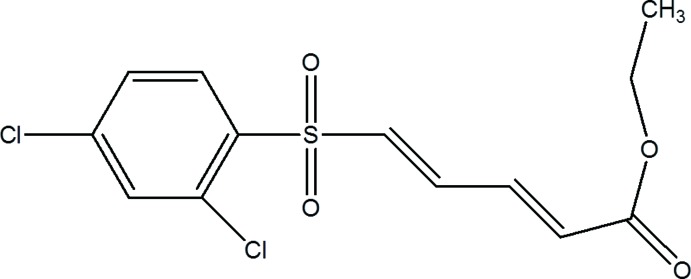


## Experimental

### 

#### Crystal data

C_13_H_12_Cl_2_O_4_S
*M* *_r_* = 335.19
Monoclinic, 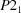

*a* = 5.773 (5) Å
*b* = 9.939 (5) Å
*c* = 13.268 (5) Å
β = 95.876 (5)°
*V* = 757.3 (8) Å^3^
*Z* = 2
Mo *K*α radiation
μ = 0.57 mm^−1^
*T* = 293 K
0.30 × 0.30 × 0.20 mm

#### Data collection

Bruker Kappa APEXII CCD diffractometer
Absorption correction: multi-scan (*SADABS*; Bruker, 2008 ▶) *T*_min_ = 0.824, *T*_max_ = 0.847
6848 measured reflections
2471 independent reflections
2314 reflections with *I* > 2σ(*I*)
*R*_int_ = 0.023

#### Refinement

*R*[*F* ^2^ > 2σ(*F* ^2^)] = 0.030
*wR*(*F* ^2^) = 0.074
*S* = 1.06
2471 reflections
209 parameters
74 restraints
H-atom parameters constrained
Δρ_max_ = 0.24 e Å^−3^
Δρ_min_ = −0.21 e Å^−3^
Absolute structure: Flack (1983 ▶), 1054 Friedel pairs
Absolute structure parameter: 0.04 (6)

### 

Data collection: *APEX2* (Bruker, 2008 ▶); cell refinement: *SAINT* (Bruker, 2008 ▶); data reduction: *SAINT*; program(s) used to solve structure: *SHELXS97* (Sheldrick, 2008 ▶); program(s) used to refine structure: *SHELXL97* (Sheldrick, 2008 ▶); molecular graphics: *ORTEP-3 for Windows* (Farrugia, 2012 ▶); software used to prepare material for publication: *SHELXL97* and *PLATON* (Spek, 2009 ▶).

## Supplementary Material

Crystal structure: contains datablock(s) I, global. DOI: 10.1107/S1600536813021429/pv2638sup1.cif

Structure factors: contains datablock(s) I. DOI: 10.1107/S1600536813021429/pv2638Isup2.hkl

Supplementary material file. DOI: 10.1107/S1600536813021429/pv2638Isup3.cml

Additional supplementary materials:  crystallographic information; 3D view; checkCIF report

## Figures and Tables

**Table 1:** Hydrogen-bond geometry (Å, °)

*D*—H⋯*A*	*D*—H	H⋯*A*	*D*⋯*A*	*D*—H⋯*A*
C7—H7⋯O1^i^	0.93	2.36	3.217 (4)	153
C9—H9⋯O1^i^	0.93	2.50	3.312 (4)	146

Symmetry code: (i) 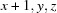.

**Fig. 1.**
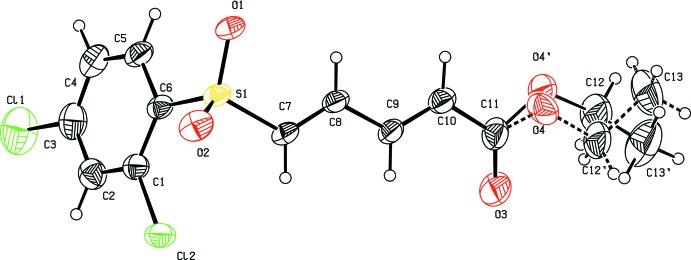
The molecular structure of the title compound, showing 30% probability displacement ellipsoids.

**Fig. 2.**
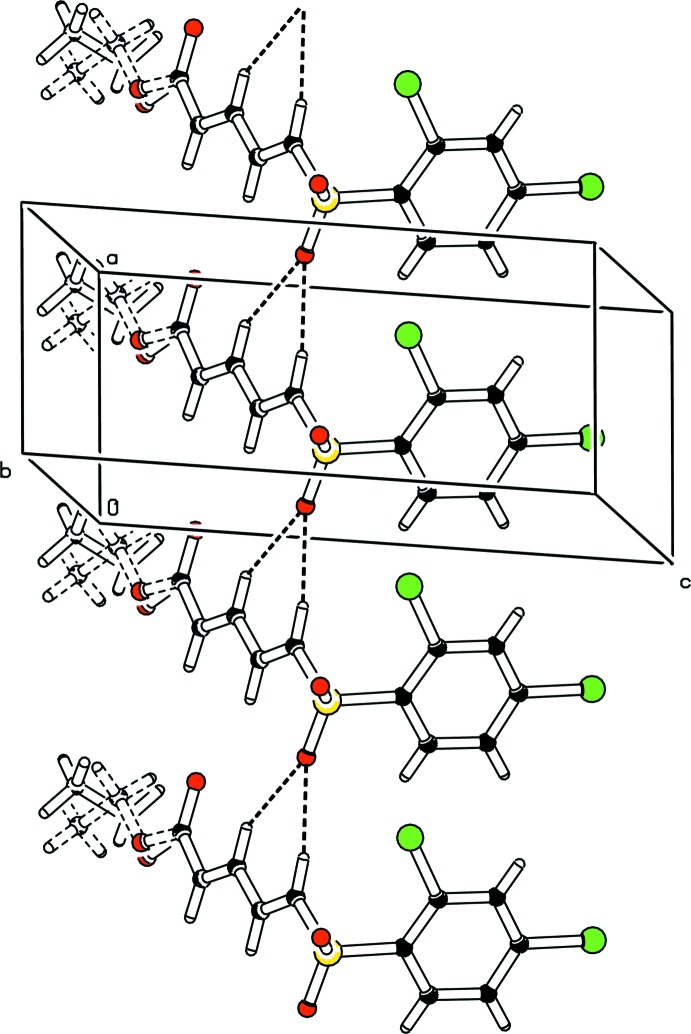
A view of the crystal packing. H atoms not involved in hydrogen bonding (dashed lines) have been omitted for clarity.

## supplementary crystallographic information

### 1. Comment

Phenyl sulfonyl containing compounds show a wide range of biological properties (De Benedetti *et al.*, 1985). The geometric parameters of the title molecule (Fig. 1) agree well with the reported values of similar structures (Sankar *et al.*, 2012; Li, 2011). Both C=C double bonds in the title compound display an E configuration. The dihedral angle between the planes (C5/C6/S1/O1) and (C1/C6/S1/O2) is 42.01 (2)°. The torsion angles C5—C6—S1—O1 and C1—C6—S1—O2 [1.1 (2)° and 50.6 (2)°, respectively] indicate synconformation of the sulfonyl moiety. The S atom exhibits significant deviation from a regular tetrahedron, with the largest deviations being seen for the O1—S1—O2 [118.12 (13)°] and C6—S1—C7 [103.3 (12)°] angles. The widening of the angles may be due to repulsive interactions between the two short S=O bonds, similar to what is observed in related structures (Chakkaravarthi *et al.*, 2008; Rodriguez *et al.*, 1995). The ethoxycarbonyl group is disordered over two conformations with site-occupancy factors of 0.739 (11) and 0.261 (11). The crystal packing is stabilized by C—H···O intermolecular interactions resulting in chains of molecules running along the *a*-axis (Tab. 1 & Fig. 2).

### 2. Experimental

Lithium bis(trimethyl silyl)amide(LHMDS) (5.4 ml, 5.7 mmol, 1.06 molar solution in THF) was added drop wise to a 258 K cooled solution of bis 2,4-dichloro phenyl sulfonyl methane (1 g, 2.3 mmol) in distilled THF (15 ml) under argon. After being stirred at 258 K for 1 h, *trans* ethyl 4-bromo crotonate (2.5 mmol) in distilled THF (5 ml) was added drop wise over a period of 10 min and allowed the reaction mixture to warm up to the room temperature over a period of 1–2 h and stirred at r.t., for 24 h. The reaction mixture was quenched with sat NH_4_Cl (20 ml) and extracted with EtOAc (2x20 ml) washed with water (2x20 ml) and sat brine (20 ml), the organic layer was dried over MgSO_4_. Evaporation of the solvent under vacuum furnished desired crude product which was purified by column chromatography on silica gel (230–400mesh) with 17–20% of EtOAc in hexane afforded the title compound as a pale yellow solid with 65% yield.

### 3. Refinement

Hydrogen atoms were placed in calculated positions with C—H ranging from 0.93 Å to 0.97 Å and refined using a riding model with fixed isotropic displacement parameters: *U*_iso_(H) = 1.5 *U*_eq_(C) for the methyl group and *U*_iso_(H) = 1.2 *U*_eq_(C) for other groups. The ethoxy carbonyl moiety was disordered over the positions O4/C12/C13:O4 /C12 /C13 with site-occupancy factors of 0.739 (11) and 0.261 (11). The absolute configuration of the reported structure was ascertained by anomalous dispersion. The refined Flack parameter (Flack, 1983) was 0.04 (6); 1417 Friedel pairs of unmerged reflections were used.

### Crystal data

**Table:**

C_13_H_12_Cl_2_O_4_S	*F*(000) = 344
*M**_r_* = 335.19	*D*_x_ = 1.470 Mg m^−^^3^
Monoclinic, *P*2_1_	Mo *K*α radiation, λ = 0.71073 Å
Hall symbol: P 2yb	Cell parameters from 8834 reflections
*a* = 5.773 (5) Å	θ = 2.1–31.2°
*b* = 9.939 (5) Å	µ = 0.57 mm^−^^1^
*c* = 13.268 (5) Å	*T* = 293 K
β = 95.876 (5)°	Block, colourless
*V* = 757.3 (8) Å^3^	0.30 × 0.30 × 0.20 mm
*Z* = 2	

### Data collection

**Table:**

Bruker Kappa APEXII CCD diffractometer	2471 independent reflections
Radiation source: fine-focus sealed tube	2314 reflections with *I* > 2σ(*I*)
Graphite monochromator	*R*_int_ = 0.023
ω and φ scan	θ_max_ = 25.0°, θ_min_ = 2.6°
Absorption correction: multi-scan (*SADABS*; Bruker, 2008)	*h* = −6→6
*T*_min_ = 0.824, *T*_max_ = 0.847	*k* = −11→8
6848 measured reflections	*l* = −15→15

### Refinement

**Table:**

Refinement on *F*^2^	Secondary atom site location: difference Fourier map
Least-squares matrix: full	Hydrogen site location: inferred from neighbouring sites
*R*[*F*^2^ > 2σ(*F*^2^)] = 0.030	H-atom parameters constrained
*wR*(*F*^2^) = 0.074	*w* = 1/[σ^2^(*F*_o_^2^) + (0.0288*P*)^2^ + 0.2277*P*] where *P* = (*F*_o_^2^ + 2*F*_c_^2^)/3
*S* = 1.06	(Δ/σ)_max_ < 0.001
2471 reflections	Δρ_max_ = 0.24 e Å^−^^3^
209 parameters	Δρ_min_ = −0.21 e Å^−^^3^
74 restraints	Absolute structure: Flack (1983), 1054 Friedel pairs
Primary atom site location: structure-invariant direct methods	Absolute structure parameter: 0.04 (6)

### Special details

**Table:**

Geometry. All e.s.d.'s (except the e.s.d. in the dihedral angle between two l.s. planes) are estimated using the full covariance matrix. The cell e.s.d.'s are taken into account individually in the estimation of e.s.d.'s in distances, angles and torsion angles; correlations between e.s.d.'s in cell parameters are only used when they are defined by crystal symmetry. An approximate (isotropic) treatment of cell e.s.d.'s is used for estimating e.s.d.'s involving l.s. planes.
Refinement. Refinement of *F*^2^ against ALL reflections. The weighted *R*-factor *wR* and goodness of fit *S* are based on *F*^2^, conventional *R*-factors *R* are based on *F*, with *F* set to zero for negative *F*^2^. The threshold expression of *F*^2^ > σ(*F*^2^) is used only for calculating *R*-factors(gt) *etc*. and is not relevant to the choice of reflections for refinement. *R*-factors based on *F*^2^ are statistically about twice as large as those based on *F*, and *R*- factors based on ALL data will be even larger.

### Fractional atomic coordinates and isotropic or equivalent isotropic displacement parameters (Å^2^)

**Table:**

	*x*	*y*	*z*	*U*_iso_*/*U*_eq_	Occ. (<1)
C1	0.5395 (4)	0.2357 (3)	0.6203 (2)	0.0521 (6)	
C2	0.5786 (6)	0.1770 (3)	0.7135 (2)	0.0667 (8)	
H2	0.7097	0.1991	0.7571	0.080*	
C3	0.4218 (6)	0.0853 (4)	0.7415 (2)	0.0741 (9)	
C4	0.2235 (6)	0.0511 (3)	0.6808 (2)	0.0714 (8)	
H4	0.1171	−0.0097	0.7028	0.086*	
C5	0.1869 (5)	0.1095 (3)	0.5862 (2)	0.0597 (7)	
H5	0.0561	0.0859	0.5430	0.072*	
C6	0.3436 (4)	0.2036 (3)	0.5546 (2)	0.0486 (6)	
C7	0.5117 (4)	0.2097 (3)	0.3680 (2)	0.0530 (6)	
H7	0.6635	0.2387	0.3858	0.064*	
C8	0.4707 (4)	0.1217 (3)	0.2951 (2)	0.0518 (6)	
H8	0.3172	0.0981	0.2747	0.062*	
C9	0.6546 (4)	0.0595 (3)	0.24492 (19)	0.0536 (6)	
H9	0.8072	0.0847	0.2656	0.064*	
C10	0.6203 (5)	−0.0303 (3)	0.1722 (2)	0.0577 (7)	
H10	0.4687	−0.0534	0.1478	0.069*	
C11	0.8169 (6)	−0.0953 (4)	0.1282 (2)	0.0722 (9)	
O1	0.0716 (3)	0.2181 (2)	0.38846 (15)	0.0669 (6)	
O2	0.3050 (4)	0.4175 (2)	0.43995 (17)	0.0713 (6)	
O3	1.0171 (4)	−0.0764 (4)	0.1560 (2)	0.1189 (12)	
Cl1	0.4769 (3)	0.00684 (14)	0.85912 (9)	0.1250 (5)	
Cl2	0.74573 (11)	0.35013 (8)	0.58707 (6)	0.0671 (2)	
S1	0.28638 (10)	0.27417 (7)	0.43281 (5)	0.05173 (18)	
O4	0.7333 (12)	−0.1885 (7)	0.0550 (6)	0.0776 (16)	0.739 (11)
C12	0.9044 (15)	−0.2721 (8)	0.0100 (6)	0.103 (2)	0.739 (11)
H12A	1.0289	−0.2963	0.0615	0.124*	0.739 (11)
H12B	0.8306	−0.3543	−0.0164	0.124*	0.739 (11)
C13	0.9999 (15)	−0.2001 (9)	−0.0710 (7)	0.137 (3)	0.739 (11)
H13A	1.1127	−0.2556	−0.0995	0.205*	0.739 (11)
H13B	1.0734	−0.1190	−0.0447	0.205*	0.739 (11)
H13C	0.8769	−0.1779	−0.1226	0.205*	0.739 (11)
O4'	0.777 (3)	−0.1422 (19)	0.0509 (18)	0.079 (4)	0.261 (11)
C12'	0.966 (3)	−0.205 (2)	0.0030 (17)	0.094 (5)	0.261 (11)
H12C	1.0634	−0.2571	0.0525	0.113*	0.261 (11)
H12D	1.0614	−0.1365	−0.0244	0.113*	0.261 (11)
C13'	0.865 (4)	−0.293 (2)	−0.0783 (15)	0.125 (7)	0.261 (11)
H13D	0.9866	−0.3274	−0.1152	0.188*	0.261 (11)
H13E	0.7566	−0.2425	−0.1233	0.188*	0.261 (11)
H13F	0.7854	−0.3662	−0.0498	0.188*	0.261 (11)

### Atomic displacement parameters (Å^2^)

**Table:**

	*U*^11^	*U*^22^	*U*^33^	*U*^12^	*U*^13^	*U*^23^
C1	0.0450 (13)	0.0456 (16)	0.0666 (17)	0.0056 (10)	0.0099 (11)	−0.0127 (12)
C2	0.0668 (19)	0.067 (2)	0.0655 (19)	0.0109 (16)	0.0055 (15)	−0.0085 (15)
C3	0.091 (2)	0.067 (2)	0.067 (2)	0.020 (2)	0.0172 (17)	0.0022 (17)
C4	0.075 (2)	0.0610 (19)	0.084 (2)	0.0022 (17)	0.0347 (17)	0.0024 (18)
C5	0.0443 (14)	0.0577 (19)	0.080 (2)	−0.0016 (11)	0.0208 (13)	−0.0074 (15)
C6	0.0369 (12)	0.0445 (14)	0.0659 (16)	0.0061 (11)	0.0119 (11)	−0.0089 (12)
C7	0.0330 (12)	0.0651 (18)	0.0621 (16)	−0.0004 (11)	0.0112 (11)	−0.0003 (14)
C8	0.0345 (12)	0.0667 (17)	0.0549 (15)	−0.0035 (11)	0.0075 (11)	0.0052 (13)
C9	0.0401 (12)	0.0696 (18)	0.0526 (14)	0.0002 (14)	0.0119 (10)	0.0025 (15)
C10	0.0445 (14)	0.0692 (19)	0.0602 (16)	0.0018 (12)	0.0088 (12)	0.0036 (15)
C11	0.0620 (19)	0.096 (3)	0.0594 (17)	0.0193 (17)	0.0110 (15)	−0.0055 (17)
O1	0.0306 (8)	0.0966 (16)	0.0740 (13)	0.0012 (9)	0.0072 (8)	−0.0091 (11)
O2	0.0631 (12)	0.0541 (13)	0.0978 (16)	0.0121 (11)	0.0133 (11)	0.0071 (12)
O3	0.0513 (14)	0.201 (3)	0.1044 (19)	0.0282 (17)	0.0099 (13)	−0.048 (2)
Cl1	0.1582 (11)	0.1325 (11)	0.0856 (7)	0.0214 (8)	0.0194 (7)	0.0369 (6)
Cl2	0.0490 (4)	0.0649 (4)	0.0873 (5)	−0.0096 (4)	0.0072 (3)	−0.0181 (4)
S1	0.0318 (3)	0.0585 (4)	0.0656 (4)	0.0040 (3)	0.0089 (2)	−0.0020 (4)
O4	0.079 (3)	0.069 (4)	0.088 (2)	−0.002 (2)	0.024 (2)	−0.027 (3)
C12	0.120 (5)	0.084 (5)	0.111 (5)	0.001 (4)	0.036 (4)	−0.033 (4)
C13	0.148 (7)	0.119 (7)	0.157 (7)	0.014 (5)	0.081 (6)	−0.010 (5)
O4'	0.084 (8)	0.062 (9)	0.090 (7)	−0.001 (6)	0.004 (6)	−0.031 (7)
C12'	0.105 (9)	0.089 (10)	0.095 (8)	−0.003 (8)	0.041 (8)	−0.039 (8)
C13'	0.164 (15)	0.109 (14)	0.104 (12)	0.009 (12)	0.019 (11)	−0.054 (11)

### Geometric parameters (Å, º)

**Table:**

C1—C2	1.366 (4)	C10—H10	0.9300
C1—C6	1.393 (4)	C11—O4'	1.13 (2)
C1—Cl2	1.735 (3)	C11—O3	1.191 (4)
C2—C3	1.363 (5)	C11—O4	1.392 (8)
C2—H2	0.9300	O1—S1	1.430 (2)
C3—C4	1.373 (5)	O2—S1	1.431 (2)
C3—Cl1	1.744 (3)	O4—C12	1.464 (6)
C4—C5	1.380 (4)	C12—C13	1.447 (9)
C4—H4	0.9300	C12—H12A	0.9700
C5—C6	1.395 (4)	C12—H12B	0.9700
C5—H5	0.9300	C13—H13A	0.9600
C6—S1	1.761 (3)	C13—H13B	0.9600
C7—C8	1.307 (4)	C13—H13C	0.9600
C7—S1	1.752 (3)	O4'—C12'	1.456 (10)
C7—H7	0.9300	C12'—C13'	1.461 (17)
C8—C9	1.449 (4)	C12'—H12C	0.9700
C8—H8	0.9300	C12'—H12D	0.9700
C9—C10	1.315 (4)	C13'—H13D	0.9600
C9—H9	0.9300	C13'—H13E	0.9600
C10—C11	1.477 (4)	C13'—H13F	0.9600
			
C2—C1—C6	121.1 (3)	O4—C11—C10	109.9 (4)
C2—C1—Cl2	117.1 (2)	O1—S1—O2	118.12 (13)
C6—C1—Cl2	121.7 (2)	O1—S1—C7	108.15 (13)
C3—C2—C1	118.7 (3)	O2—S1—C7	109.99 (13)
C3—C2—H2	120.7	O1—S1—C6	107.18 (12)
C1—C2—H2	120.7	O2—S1—C6	109.29 (14)
C2—C3—C4	122.9 (3)	C7—S1—C6	103.03 (12)
C2—C3—Cl1	118.5 (3)	C11—O4—C12	117.5 (6)
C4—C3—Cl1	118.6 (3)	C13—C12—O4	110.3 (6)
C3—C4—C5	118.0 (3)	C13—C12—H12A	109.6
C3—C4—H4	121.0	O4—C12—H12A	109.6
C5—C4—H4	121.0	C13—C12—H12B	109.6
C4—C5—C6	120.8 (3)	O4—C12—H12B	109.6
C4—C5—H5	119.6	H12A—C12—H12B	108.1
C6—C5—H5	119.6	C12—C13—H13A	109.5
C1—C6—C5	118.4 (3)	C12—C13—H13B	109.5
C1—C6—S1	123.1 (2)	H13A—C13—H13B	109.5
C5—C6—S1	118.5 (2)	C12—C13—H13C	109.5
C8—C7—S1	121.3 (2)	H13A—C13—H13C	109.5
C8—C7—H7	119.3	H13B—C13—H13C	109.5
S1—C7—H7	119.3	C11—O4'—C12'	118.9 (17)
C7—C8—C9	122.7 (2)	O4'—C12'—C13'	108.6 (13)
C7—C8—H8	118.7	O4'—C12'—H12C	110.0
C9—C8—H8	118.7	C13'—C12'—H12C	110.0
C10—C9—C8	124.4 (2)	O4'—C12'—H12D	110.0
C10—C9—H9	117.8	C13'—C12'—H12D	110.0
C8—C9—H9	117.8	H12C—C12'—H12D	108.4
C9—C10—C11	121.5 (3)	C12'—C13'—H13D	109.5
C9—C10—H10	119.2	C12'—C13'—H13E	109.5
C11—C10—H10	119.2	H13D—C13'—H13E	109.5
O4'—C11—O3	116.5 (10)	C12'—C13'—H13F	109.5
O3—C11—O4	125.2 (4)	H13D—C13'—H13F	109.5
O4'—C11—C10	116.6 (10)	H13E—C13'—H13F	109.5
O3—C11—C10	124.7 (3)		
			
C6—C1—C2—C3	0.1 (4)	C9—C10—C11—O4	178.3 (4)
Cl2—C1—C2—C3	−179.1 (2)	C8—C7—S1—O1	−2.3 (3)
C1—C2—C3—C4	−1.2 (5)	C8—C7—S1—O2	−132.6 (2)
C1—C2—C3—Cl1	178.1 (2)	C8—C7—S1—C6	110.9 (3)
C2—C3—C4—C5	2.1 (5)	C1—C6—S1—O1	−179.8 (2)
Cl1—C3—C4—C5	−177.3 (2)	C5—C6—S1—O1	1.1 (2)
C3—C4—C5—C6	−1.8 (4)	C1—C6—S1—O2	−50.6 (2)
C2—C1—C6—C5	0.1 (4)	C5—C6—S1—O2	130.3 (2)
Cl2—C1—C6—C5	179.29 (19)	C1—C6—S1—C7	66.3 (2)
C2—C1—C6—S1	−179.0 (2)	C5—C6—S1—C7	−112.8 (2)
Cl2—C1—C6—S1	0.2 (3)	O4'—C11—O4—C12	73 (3)
C4—C5—C6—C1	0.8 (4)	O3—C11—O4—C12	1.3 (9)
C4—C5—C6—S1	180.0 (2)	C10—C11—O4—C12	−174.4 (5)
S1—C7—C8—C9	−175.6 (2)	C11—O4—C12—C13	−82.9 (10)
C7—C8—C9—C10	179.3 (3)	O3—C11—O4'—C12'	15 (2)
C8—C9—C10—C11	−176.5 (3)	O4—C11—O4'—C12'	−105 (4)
C9—C10—C11—O4'	−159.7 (13)	C10—C11—O4'—C12'	178.7 (15)
C9—C10—C11—O3	2.6 (6)	C11—O4'—C12'—C13'	163 (2)

### Hydrogen-bond geometry (Å, º)

**Table:**

*D*—H···*A*	*D*—H	H···*A*	*D*···*A*	*D*—H···*A*
C7—H7···O1^i^	0.93	2.36	3.217 (4)	153
C9—H9···O1^i^	0.93	2.50	3.312 (4)	146

Symmetry code: (i) *x*+1, *y*, *z*.
